# Near-Infrared Responsive Phase-Shifted Nanoparticles for Magnetically Targeted MR/US Imaging and Photothermal Therapy of Cancer

**DOI:** 10.3389/fbioe.2020.599107

**Published:** 2020-11-16

**Authors:** Yan Xu, Wang Li, Sijie Chen, Biying Huang, Wenjing Pei, Chengcheng Niu

**Affiliations:** ^1^Department of Ultrasound Diagnosis, The Second Xiangya Hospital, Central South University, Changsha, China; ^2^Research Center of Ultrasonography, The Second Xiangya Hospital, Central South University, Changsha, China

**Keywords:** magnetically targeted, MR/US imaging, perfluorocarbon, phase shifted, photothermal therapy

## Abstract

Accurate diagnosis, providing guidance for early treatment, can greatly improve the survival rate of cancer patients. However, there are still some difficulties with the existing diagnostic technology and early treatment methods. Here, near-infrared responsive phase-shifted nanoparticles (NRPNs) have been designed for magnetically targeted MR/US imaging and photothermal therapy of tumors. In this study, we fabricated a multifunctional polymer nanoparticle encapsulating indocyanine green (ICG), magnetic Fe_3_O_4_ nanoparticles and perfluoropentane (PFP). Under laser irradiation, the NRPNs, which trigger a phase-shifted expansion effect due to the quick conversion from light to heat by ICG and Fe_3_O_4_, can be used for ultrasound (US) imaging. At the same time, such nanoparticles can kill cancer cells via photothermal therapy (PTT). As a kind of negative enhancement agent, magnetic Fe_3_O_4_ nanoparticles in NRPNs showed high spatial resolution in MR imaging. Moreover, with the help of the magnetic field, the NRPNs nanoparticles showed high cellular uptake and high tumor accumulation, indicating their magnetic targeting property without biosafety concerns. Therefore, we present a strategy for magnetically targeted MR/US imaging guided photothermal therapy for the accurate diagnosis and efficient treatment of tumors.

## Introduction

Cancer is one of the major diseases associated with human mortality. With the increasing numbers of cancer patients worldwide, cancer has become a major public health problem threatening human health (Siegel et al., 2020). Earlier and more accurate diagnosis of cancer, to provide guidance for early treatment, can greatly improve the survival rate of cancer patients (Hussain and Nguyen, 2014). For more accurate biological details of the solid tumors, multimodal imaging, which integrates various of imaging techniques, such as ultrasound (US), magnetic resonance (MR), computed tomography (CT), positron emission tomography (PET) or optical imaging, has increasingly attracted much attention.

Recent studies have shown that liquid perfluorocarbon (PFC) could be used as a phase-shift enhancement agent encapsulated in nanoparticles (NPs) due to its low boiling point. These encapsulated PFC NPs could produce excellent contrast for US imaging by phase-transition of microbubbles via optical droplet vaporization (ODV) (Eric, 2011; Hannah et al., 2014; Santiesteban et al., 2017). In addition, owing to their small size (less than 700 nm), the encapsulated PFC NPs can accumulated in the tumor site by passing through the endothelial cells gaps in the blood vessels of tumors (Jian et al., 2014; Santiesteban et al., 2019). By encapsulating optical absorbing materials [i.e., organic (Zhu et al., 2018; Chen et al., 2019) and inorganic compounds (Li et al., 2018; Guan et al., 2019)] into the PFC NPs, the NPs can quickly transfer to microbubbles under NIR laser irradiation exposure induction. Once triggered into microbubbles, such NPs can produce excellent contrast-enhanced US imaging as well (Jian et al., 2014; Sun et al., 2014). Indocyanine green (ICG), a near infrared (NIR) organic dye, was approved by the US Food and Drug Administration (FDA) (Sheng et al., 2014) due to its very low rate of side effects (Sakka, 2007), and it can absorb light in the NIR region (Hu et al., 2016) and convert it into heat (Yan and Qiu, 2015; Li et al., 2016). This results in photothermal therapy (PTT) with the temperature exceeding 42°C, which can promote the death of cancer cells and inhibit tumor growth. However, ICG also suffers from several inherent drawbacks limiting its applications, such as its instability in aqueous solution (Saxena et al., 2003), rapid liver elimination (Ott, 1998), and temperature- and light-dependent optical properties (Ma et al., 2013). Moreover, ICG cannot actively target tumors (Yan and Qiu, 2015). Therefore, how to overcome these drawbacks and promote a targeting performance for enhancing the phototherapy of ICG is challenging work.

Magnetic iron oxide (Fe_3_O_4_) NPs have been widely studied for the treatment of cancer owing to their unique characteristics, such as acting as the magnetic hyperthermia or photothermal agents to increase temperature in response to magnetic induction or laser irradiation (Espinosa et al., 2016; Dadfar et al., 2019). Fe_3_O_4_ is typically used as an MR imaging contrast agent because of its typical darkening property due to the short transverse relaxation time (T2) of protons (Zeng et al., 2013; Wang et al., 2014). Moreover, Fe_3_O_4_ can be targeted to the tumor region with the help of a magnet, resulting in tissue-specific accumulation (Cheng et al., 2012; Zhu et al., 2013).

**FIGURE 1:**
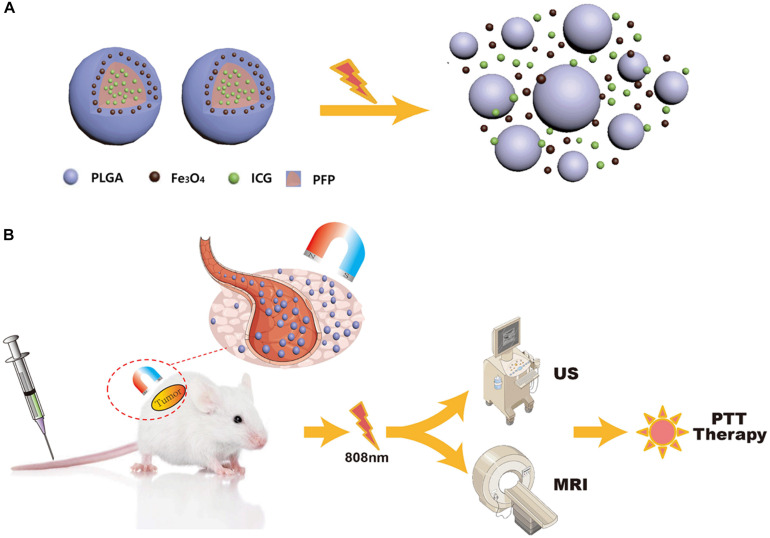
**(A)** The optical droplet vaporization process of the NRPNs. **(B)** Schematic Illustration of the NRPNs used for magnetically targeted dual-modal imaging guided photothermal therapy of tumor.

In this study, we synthesized near-infrared responsive phase-shifted nanoparticles (NRPNs) by incorporating ICG, PFP and Fe_3_O_4_ NPs into poly lactic-co-glycolic acid (PLGA) shells with a magnetic field for dual-modal enhancement of US/MR imaging via a phase-shift expansion and PTT treatment by NIR laser (Figure 1) based on the following considerations: (i) Previously, NRPNs were used as an effective therapeutic agent for tumor ablation via PTT (Niu et al., 2017). (ii) After NIR laser irradiation, Fe_3_O_4_ NPs and ICG can absorb NIR light and transfer it into heat, triggering the liquid-to-gas transition of PFP and developing a specific “nano-to-micro” phase-transformation strategy for contrast enhanced US (Xu et al., 2017; Wang et al., 2018). (iii) Dual-modal imaging of NRPNs, which integrates MR and US imaging, can offer imaging guidance for PTT of tumors. (iv) With the driving action of the magnetic field, more NRPNs can accumulate in the tumor region and will be more effective for the diagnosis and treatment of the tumor.

## Materials and Methods

### Preparation of the NRPNs

The synthesis of the NRPNs followed the previous reports (Niu et al., 2017). First, 400 μL liquid PFP and 400 μL ICG solution (1 mg ICG dissolved in 100 μL of deionized water) were mixed, and then emulsified with an ultrasonic sonicator for 30 s. PLGA (100 mg) and Fe_3_O_4_ NPs (200 μL) were dissolved in 3 mL chloroform. Then, the PFP and ICG mixture was added into the chloroform with a second emulsification for 1 min. Subsequently, 15 mL cold 4% PVA was added to the above emulsified solution for a third emulsification for 2 min. The resulting emulsion was volatilized by evaporation for 2 h. At last, the NRPNs were washed with deionized water 3 times and stored at 4°C in the dark until further use. All of the operations were performed in an ice bath and in the dark. The same procedure was used to prepare the PLGA NPs without ICG, Fe_3_O_4_ and PFP, PFP/ICG/PLGA NPs without Fe_3_O_4_ and PFP/ICG/PLGA NPs without Fe_3_O_4_. These NPs were used as controls.

For cell uptake experiments, the fluorescent DiI labeled NRPNs was prepared. PLGA (100 mg), Fe_3_O_4_ NPs (200 μL) and 1 mg DiI were dissolved in 3 mL chloroform, 400 μL liquid PFP and 400 μL ICG solution (1 mg ICG dissolved in 100 μL of deionized water) were mixed, and then emulsified with an ultrasonic sonicator for 30 s. Then PFP and ICG mixture was added into to the chloroform with second emulsification for 1 min. Subsequently, 15 mL cold 4% PVA was added to the above emulsified solution with the third emulsification for 2 min. The resulting emulsion was volatilized by evaporation for 2 h. At last, the NRPNs was washed with deionized water 3 times and stored at 4°C in dark for further use.

### Characterization

The morphological characteristics of the NRPNs was detected by scanning electron microscopy (SEM). Structural characterization and the existence of Fe_3_O_4_ NPs were measured using transmission electron microscopy (TEM). Size distributions and zeta potential were analyzed using a Malvern size analyzer. The encapsulated iron amount in the NRPNs was calculated by atomic absorption spectrometry. The UV–Vis-NIR absorption spectra of the NRPNs was detected by a UV–vis-NIR spectrophotometer to confirm the existence of ICG. The ICG encapsulation efficiency and loading content were calculated thus:

Encapsulationefficiency(%)=(MassofalloftheencapsulatedICGintheNRPNs)/(Mass⁢of⁢all⁢of⁢the⁢added⁢ICG)×100%

Loadingcontent(%)=(MassofalloftheencapsulatedICGintheNRPNs)/(Mass⁢of⁢the⁢NRPNs)×100%

### Temperature Elevation and NIR-Responsive Phase-Shift for US Imaging With NRPNs

Two hundred μL of various concentrations of the NRPNs (0, 2.5, 5.0, and 7.5 μg/mL ICG), ICG/PFP/PLGA NPs (5.0 μg/mL ICG), Fe_3_O_4_/PFP/PLGA NPs (the amount of Fe_3_O_4_ was equivalent to the NRPNs at 5.0 μg/mL ICG with a concentration of 8.0 μg/mL Fe) and PFP/PLGA were set in a 96-well plate and irradiated by an 808 nm NIR laser for 10 min. The power of the laser was 1 W/cm^2^. The temperature of the NRPNs was measured under NIR laser irradiation by an infrared thermal imaging camera. The temperatures of the NPs were measured every 30 s. The phase-shift of the NRPNs was observed with TEM and an optical microscope.

To examine the vaporization process of US imaging, approximately 1 mL of the NRPNs suspension was injected into an agar-gel model. The NRPNs suspension was irradiated by a laser. The power of the laser was 1 W/cm^2^. Then, an US apparatus was used to scan the suspension for B-mode and contrast-enhanced US (CEUS) mode imaging.

### *In vitro* Stability Study of NRPNs

To access the stability of the nanoparticles, the experiments were conducted by measuring the DLS diameters of the NRPNs in PBS or 10% FBS (HyClone) at 37°C every 24 h for 7 days.

### *In vitro* MR Imaging

To assess the *in vitro* MR imaging, different Fe_3_O_4_ concentrations (0.0, 0.1, 0.2, 0.3, 0.4, and 0.5 mg/mL) of the NRPNs suspension were added to 2 mL Eppendorf tubes for MR imaging using a 3.0 T MRI apparatus. The T2-weighted images (T2 WI) signal intensity (SI) at each concentration was measured.

### Cell Experiments

For the *in vitro* magnetic targeting efficacy study of the NRPNs, a cellular uptake study was assessed by confocal laser scanning microscopy (CLSM). The MCF-7 cells (1 × 10^5^) were planted into glass-bottomed Petri dishes and cultured with 200 μL serum-free medium containing the DiI labeled NRPNs nanoparticles (0.2 mg/mL) for 2 h with or without a magnet. Cells without nanoparticles were treated as control group. The maximum magnet strength was 6.0 Gs. The thickness of the petri dish was about 1 mm, and the depth of the liquid in each dish was 3 mm. For a distance of 1 mm from the magnet, the maximum magnetic field strength was 5.5 Gs. For a distance of 4 mm from the magnet, the maximum magnetic field strength was 4.8 Gs. Then, each well was washed with PBS 3 times and the cells were stained with DAPI for 10 min before CLSM imaging.

Cell counting kit (CCK-8) assays were used to assess the *in vitro* cytotoxicity of the NRPNs and to inspect the photothermal efficiency under the magnetic field. MCF-7 cells (1 × 10^4^ per well) were seeded onto 96-well plates and incubated for 24 h. Subsequently, the NRPNs with a serial concentration of 0, 200, 400, 600, 800, and 1000 μg/mL were incubated with the cells for 6 h with or without a magnet. The maximum magnet strength was 6.0 Gs. The thickness of the 96-well plate was about 1 mm, and the depth of the liquid in each well was 3 mm. For a distance of 1 mm from the magnet, the maximum magnetic field strength was 5.5 Gs. For a distance of 4 mm from the magnet, the maximum magnetic field strength was 4.8 Gs. Afterward, these wells continued to culture until 24 h, and then washed with PBS and irradiated by an NIR laser for 5 min. The power of the laser was 1.0 W/cm^2^. Finally, a CCK-8 assay was used to measure the cell viability.

The apoptosis of MCF-7 cells was evaluated by Calcein-AM/PI double staining kit. Cells were seeded on a 6-well plate and then incubated with PBS or NRPNs for 2 h. The NRPNs groups were treated with or without a magnet and the concentration of NRPNs was 0.2 mg/mL in each well. The maximum magnet strength was 6.0 Gs. The thickness of the 6-well plate was about 1 mm, and the depth of the liquid in each well was 3 mm. For a distance of 1 mm from the magnet, the maximum magnetic field strength was 5.5 Gs. For a distance of 4 mm from the magnet, the maximum magnetic field strength was 4.8 Gs. After co-incubation with different treatments, the medium was removed and washed with PBS for 3 times. The laser irradiation was at the intensity of 1 W/cm^2^ for 5 min. Then the Calcein-AM/PI double staining kit was added to each well in the dark at an appropriate concentration for 15 min at room temperature. After that, the cells were thoroughly washed and imaged through a fluorescence microscope.

### Animal Studies

All animal experiments procedures were approved by the Ethics Committee of the Second Xiangya Hospital of Central South University. Female 4-week-old BALB/c mice were bred at the department of Laboratory Animals of Central South University. To establish the tumor model, the mice were injected subcutaneously with 1 × 10^6^ 4T1 cells into their right flanks. The size of the tumors was observed for 2 weeks. The volume of the tumors that achieved 60 mm^3^ were used for the experiment.

### *In vivo* Toxicity and Biodistribution Studies

For the *in vivo* biological toxicity, 200 μL of NRPNs solutions (20 mg/kg) was intravenously injected into 5 female BALB/c mice and 200 μL saline was intravenously injected into the other 5 female BALB/c mice as controls. These mice were sacrificed 14 days later. Then, the tissues of the brain, heart, liver, spleen, kidney and lung of each mouse were fixed with 4% formaldehyde solution and observed by H&E.

For assessment of the biodistribution and the magnetic targeting effect of the NRPNs, 200 μL of the NRPNs solutions (20 mg/kg) was intravenously injected into 10 tumor-bearing mice. Five mice were treated with magnetic targeting, the other 5 mice were treated without magnetic targeting. A magnet was placed next to the tumor region for 8 h for targeting. The maximum magnet strength was 6.0 Gs. At the 0, 1, 8, and 24 h time point, the fluorescence of each tumor was obtained with a Xenogen IVIS Spectrum *in vivo* imaging system. After 24 h, all of the tumors and major organs (brain, heart, liver, spleen, kidney, and lung) of the mice were *ex vivo* imaged by the fluorescence system.

### *In vivo* MR Imaging

In the *in vivo* MRI experiments, 200 μL of the NRPNs solutions (20 mg/kg) was intravenously injected into 10 tumor-bearing mice. Five mice were treated with magnetic targeting, the other 5 mice were treated without magnetic targeting. A magnet was placed next to the tumor region for 8 h for the targeting. The maximum magnet strength was 6.0 Gs. The MRI images of the tumors were captured with a 3.0 T MRI Skyra scanner before and 24 h after injection. Finally, the SI within the ROI of the MRI images were measured.

### *In vivo* US Imaging

In the *in vivo* US experiments, 200 μL of the NRPNs solutions (20 mg/kg) was intravenously injected into 10 tumor-bearing mice. Five mice were treated with magnetic targeting, the other 5 mice were treated without magnetic targeting. A magnet was placed next to the tumor for 8 h for targeting. The maximum magnet strength was 6.0 Gs. The mice were scanned by a Siemens S3,000 US scanner 24 h after injection. Then, the tumors were irradiated by an NIR laser for 6 min (808 nm, 1.0 W/cm^2^). US images in B-mode and CEUS mode were obtained by a Siemens ultrasonography machine before and after laser irradiation.

### *In Vivo* Anticancer Treatment Performance

Tumor-bearing mice were randomly divided into five groups (*n* = 3 for per group): (1) saline with laser irradiation, (2) only the NRPNs, (3) the NRPNs with laser irradiation but without magnetic targeting, (4) ICG/PFP/PLGA NPs with magnetic targeting and laser irradiation and (5) the NRPNs with magnetic targeting and laser irradiation. The dose of the NRPNs (20 mg/kg), saline or ICG/PFP/PLGA NPs (20 mg/kg) was 200 μL per mice by an intravenous injection. A magnet was placed next to the tumor for 8 h. The maximum magnet strength was 6.0 Gs. After 24 h, the tumors were subjected to laser irradiation for 10 min. The power of the laser was 1 W/cm^2^. At the same time, the temperature of the tumor region was measured every 30 s. Tumor volumes were monitored by a caliper every 2 days. The tumor volume was calculated as: *V* = L × W^2^/2 (*L* = the length of the tumor and *W* = the width of the tumor), and 14 days after laser irradiation, the tumors were collected and fixed with 4% formaldehyde solution for immunohistochemistry.

### Statistical Analysis

All experimental data were expressed as the mean ± SD. Comparisons of two groups were analyzed by Student’s *t-*test and multiple groups were analyzed by two-way analysis using SPSS 18.0. *p* < 0.05 was considered significant.

## Results and Discussion

### Characterization

**FIGURE 2:**
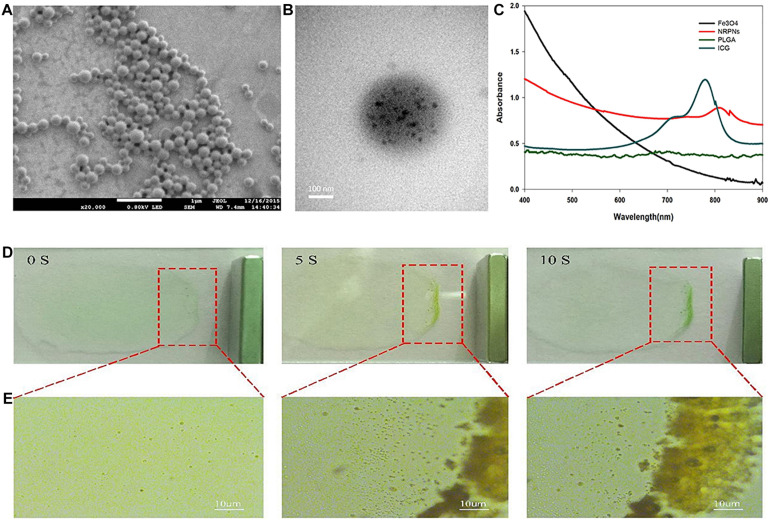
**(A)** The SEM image of the NRPNs. **(B)** The TEM image shows the black Fe_3_O_4_ NPs embedded in the PLGA shell. **(C)** UV–vis–NIR absorption spectra of free Fe_3_O_4_, free ICG, PLGA NPs, and NRPNs. **(D)** Photograph, and **(E)** optical microscopy images of NRPNs solution (10 mg/mL) in a slide with an external magnetic field.

The NRPNs were produced using our group’s previously published method with a different concentration of ICG (Niu et al., 2017). SEM images revealed that the NRPNs had a smooth and uniform spherical morphology (Figure 2A). The TEM images showed that the multiple Fe_3_O_4_ NPs were well encapsulated within the PLGA shells (Figure 2B). The nanoparticle average diameter was 315.29 ± 15.66 nm. The zeta potential was −21.1 ± 7.4 mV. The absorption spectra of the different components are shown in Figure 2C, revealing that ICG and Fe_3_O_4_ were successfully loaded onto the NRPNs. The EE of ICG was above 30.25 ± 5.13% and the LC of ICG was above 1.25 ± 0.52%. The Fe content was 97.30 ± 2.82 μg/mL.

A magnetic model was used to assess the magnetic response of the NRPNs. As shown in Figure 2D, the NRPNs were dispersed in water on the slide. A magnet was placed next to the slide. As the time increased, the solution of the NRPNs became increasingly colorless and most of the NRPNs were clustered together near the magnet from 0 to 10 s. Under the light microscope, the NRPNs nanoparticles were observed to aggregate and move toward the magnet (Figure 2E).

### Temperature Eelevation and Phase Transition Induced by NIR Laser Irradiation

**FIGURE 3:**
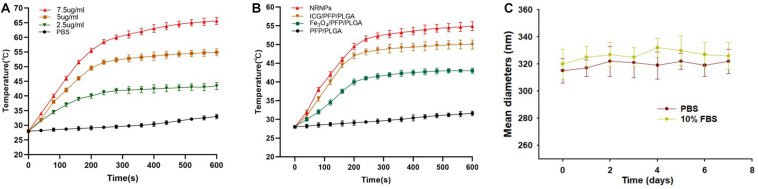
**(A)** The temperature curves of the NRPNs with different concentration of laser irradiation at 1 W/cm^2^. **(B)** The temperature curves of different nanoparticles with laser irradiation at 1 W/cm^2^. **(C)** Size distributions of NRPNs in PBS or 10% FBS for 7 days.

To study the temperature elevation, an 808 nm NIR laser was used to irradiate the NRPNs for 10 min. With the laser irradiation, the temperature of the different concentrations of NRPNs increased rapidly from 43.4°C (at 2.5 mg/mL) to 65.6°C (at 7.5 mg/mL) (Figure 3A). This finding indicates that the temperature is positively correlated with the concentration of the NRPNs. As shown in Figure 3B, the temperature of the NRPNs (at 5.0 mg/mL) rapidly exceeded 54.9°C, while the PLGA/PFP/ICG NPs without Fe_3_O_4_ and the Fe_3_O_4_/PFP/PLGA NPs without ICG only achieved 50°C and 43°C, respectively. This finding indicates that the Fe_3_O_4_ and ICG worked together to produce the NIR-responsive temperature increase and that the NRPNs reached up to 42°C, which is the temperature point necessary for triggering cancer-cell damage. Meanwhile, with an increasing temperature up to 50°C, the liquid PFP of the PLGA nanoparticles could convert into a gaseous state with a phase-change. That was very important for US imaging.

The size distributions of NRPNs stored in PBS or 10% FBS at 37°C for 7 days were measured to investigate the colloidal stability. As shown in Figure 3C, there was no significant change in the particle size distribution over time. Therefore, the nanoparticles exhibited good colloidal stability and could be used in subsequent experiments.

**FIGURE 4:**
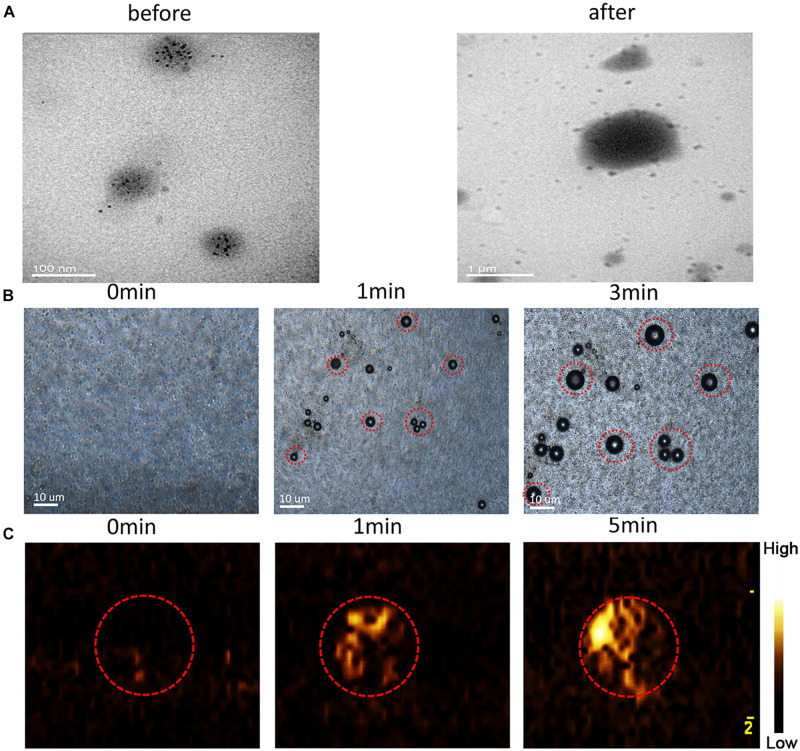
**(A)** TEM images of the NRPNs under 808 nm NIR irradiation (1.0 W/cm^2^, 5 min). **(B)** Images of phase-shift of the NRPNs with the laser irradiation by microscopy (1.0 W/cm^2^, 3 min). **(C)** The CEUS imaging of the NRPNs under the laser irradiation (1.0 W/cm^2^, 5 min).

Transmission electron microscopy analysis revealed the morphological and size changes of the NRPNs before and after laser irradiation (Figure 4A). After laser irradiation, the particle size increased significantly and Fe_3_O_4_ was released from the nanoparticles. The optical microscopy images further revealed that no microbubbles were observed before NIR irradiation (Figure 4B). After 1 min of irradiation, some of the NRPNs started to expand. When the time reached 3 min, the size of the NPs increased. A high efficiency of converting to microbubbles from the NRPNs after laser irradiation is important for US imaging. As the time increased, increasing number of microbubbles were produced, but we could not observe the same microbubbles changing since they moved to the upper level via their buoyancy.

At the same time, the NRPNs solutions were subjected to US imaging in CEUS mode (Figure 4C) at different times after laser irradiation. As the time increased under laser irradiation, the echo intensity of CEUS imaging became much stronger. These findings indicated that the size of the NRPNs was significantly increased after laser irradiation (1 W/cm^2^, 5 min) and microbubble production resulting from the phase-change phenomenon was consistent with the strong CEUS echogenicity in the US imaging.

### *In vitro* US/MR Imaging

**FIGURE 5:**
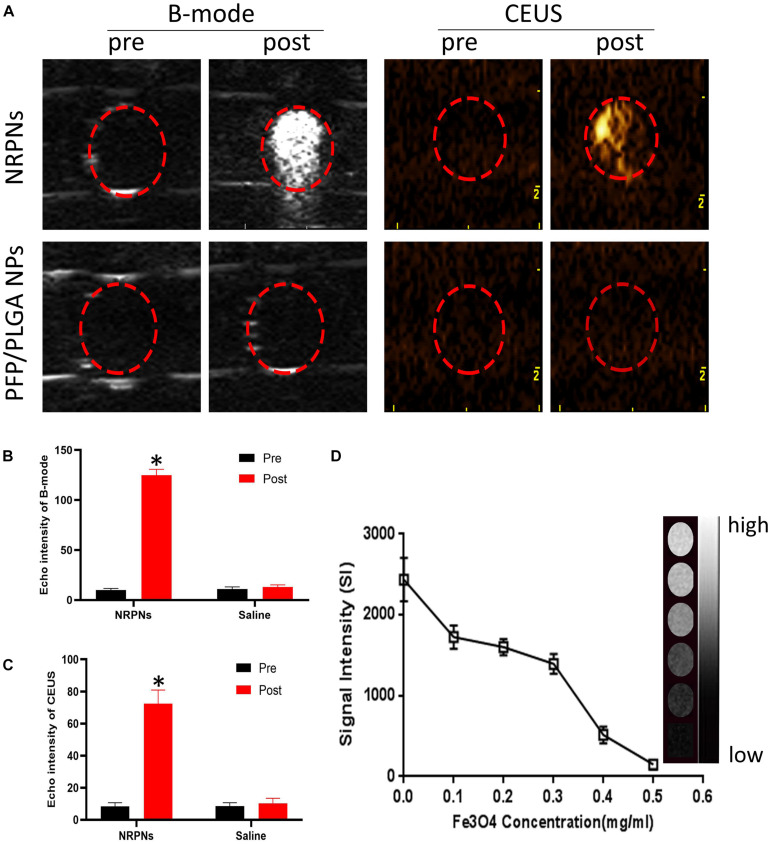
**(A)** B-mode and CEUS imaging of the NPs with the laser irradiation (1 W/cm^2^, 5 min). Echo intensity in B-mode **(B)** and CEUS **(C)** mode of NPs with the laser irradiation (1 W/cm^2^, 5 min). **(D)** T2 WI SI curve of the NRPNs at different Fe_3_O_4_ concentrations. The right gray bar indicates the T2 MRI image at different Fe_3_O_4_ concentrations. The difference is statistically significant (^∗^*p* < 0.05).

To obtain more accurate biological details of solid tumors, dual-modality imaging has been studied widely. As shown in Figure 5A, there were no enhancement signals before and after NIR irradiation of the PFP/PLGA nanoparticles without ICG and Fe_3_O_4_. However, in the NRPNs group, when the NRPNs were irradiated by an 808 nm laser, significant enhancement in the B-mode and CEUS were observed. As shown in Figures 5B,C, the echo intensity of the B-mode and CEUS in the NRPNs group was significantly increased after laser irradiation compared to that in the PFP/PLGA nanoparticles group (^∗^*p* < 0.05). These results further demonstrated that with the quick conversion from light energy to heat by ICG and Fe_3_O_4_, the PFP vaporization process was triggered, and then the NRPNs can be used for the enhancement of US imaging.

In the *in vitro* experiment, the MR imaging capabilities of the NRPNs were examined using a 3.0 T MR scanner. As shown in Figure 5D, the NRPNs negatively enhanced the T2 weighted MR images. The T2 weighted MR SI decreased with the increasing Fe_3_O_4_ concentration. These results indicated that in addition to US imaging, the NRPNs could also effectively serve as an MRI negative contrast agent.

### The Magnetically Targeting Performance and Cytotoxicity in Cells

**FIGURE 6:**
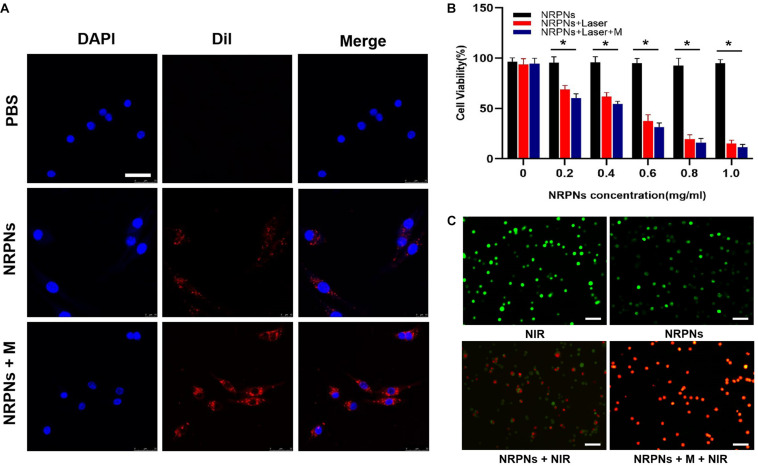
**(A)** Cellular uptake of the NRPNs with or without a magnet (scale bar, 20 μm). **(B)** Cell viability of MCF-7 cells with different concentrations of the NRPNs after 808 nm laser irradiation (1 W/cm^2^, 5 min) with or without a magnet. The difference is statistically significant (*p < 0.05). **(C)** calcein/PI staining of MCF-7 cells after different treatments: (1) NIR; (2) NRPNs; (3) NRPNs + NIR; (4) NRPNs + M + NIR (scale bar, 50 μm).

The magnetically targeting performance and cytotoxicity of the NRPNs were observed in cell experiments. The NRPNs were co-incubated with MCF-7 cells for 2 h, and divided into three groups: (1) PBS; (2) without the magnet (NRPNs) and (3) with the magnet (NRPNs + M). As shown in Figure 6A, a significantly red fluorescence appeared around the nucleus of the MCF-7 cells in the NRPNs + M group, only a little red fluorescence showed around the MCF-7 cells in the NRPNs, while no red fluorescence was observed in the PBS group. This finding demonstrated that with the help of a magnet, more NRPNs gathered around the cells, promoting their endocytosis.

Our previous experiments demonstrated low cytotoxicity and an excellent PTT effect of similar NPs (Niu et al., 2017). The same results were achieved in our current experiments. At the same NRPNs concentrations, the cell viability with laser irradiation was significantly reduced compared to that without irradiation (^∗^*p* < 0.05). In addition, with magnetic targeting, more NRPNs were attracted around the cells. The MCF-7 cells with magnet targeting showed an obvious decrease in cell viability after irradiation, compared to non-magnetic targeting (Figure 6B, ^∗^*p* < 0.05). This finding demonstrated an excellent endocytosis ability of the NRPNs by MCF-7 cells and resulted in magnetically targeted PTT.

Then, the tumor killing effect of NRPNs was observed directly by using calcein/PI staining. The cells were divided into 4 groups: (1) NIR, (2) NRPNs, (3) NRPNs + NIR, (4) NRPNs + M + NIR. We stained live and dead with calcein (green fluorescence) and PI (red fluorescence) separately. As shown in Figure 6C, the MCF-7 cells of the NIR group (1) and NRPNs group (2), emitted pure green fluorescence. After the NIR irradiation, red fluorescence could be observed to different extents. A lot of dead cells were observed in the NRPNs + NIR group (3), while almost all cells were dead in the NRPNs + M + NIR group, further suggesting that the tumor cells can be effectively killed by magnetic targeting, which promotes the cells to contact with more NPs and then uptake them.

### *In vivo* Biodistribution Studies and Biotoxicity Evaluation

**FIGURE 7:**
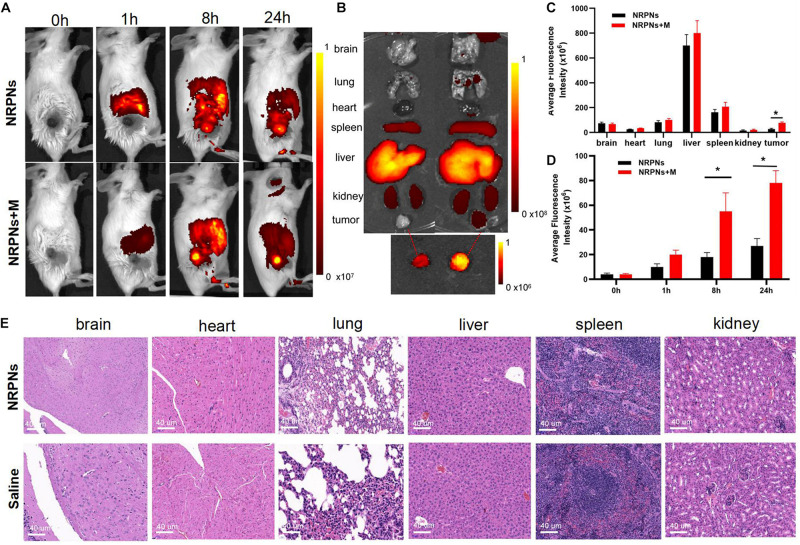
**(A)** Biodistribution of NPs in tumor-bearing mice at different time points by *in vivo* fluorescent imaging. **(B)** Fluorescence imaging of major organs and tumors at 24 h after injection of the NRPNs. **(C)** Averaged fluorescence intensity of major organs and tumors at 24 h after injection of the NRPNs. **(D)** Averaged fluorescence intensity of tumors at different time points. **(E)** H&E-staining images of major organs collected from the NRPNs and saline groups. The difference is statistically significant (*p < 0.05).

To quantify the biodistribution and the magnetically targeting performance of the NRPNs in tumor-bearing mice, fluorescence imaging was performed. With or without magnetic targeting, tumor fluorescence images were obtained at different times before and after injection with the NRPNs. As shown in Figure 7A, before administration, there were no red fluorescent signals in the tumor region or the major organs in tumor-bearing mice of the NRPNs + M group and the NRPNs group. The biodistribution of the fluorescent signals in the major organs and tumors were analyzed at 1, 8, and 24 h after injection of the NRPNs.

In the major organs, especially the reticuloendothelial system of the liver and spleen, accumulation of the NRPNs peaked at 8 h post injection, and the NRPNs content in these organs decreased after 24 h (Figure 7A). The NRPNs accumulation in the tumor region remained until 24 h post injection (Figures 7B,C), suggesting that with the help of a magnet, more NRPNs were clustered in the tumor. In the absence of the magnetic field, there were fewer red fluorescent signals of the NRPNs in the control group (Figure 7D, ^∗^*p* < 0.05), which means lower tumor accumulation in the tumor region. These findings indicated that magnet targeting provides a promising physical strategy for tumor targeting and the magnetically targeting NPs are a potential treatment platform for dual-model imaging and PTT of tumors.

In light of the biodistribution studies and the 24 h blood retention time *in vivo*, it was necessary to further assess the biotoxicity of the NRPNs *in vivo*. As shown in Figure 7E, there were no noticeable changes in inflammation or abnormal histomorphology in these major organs (heart, liver, spleen, lung, and kidney) of the mice after injection with the NRPNs, compared with the saline control group. These findings indicate that injection of the NRPNs was biosafe and they have low potential toxicity *in vivo*.

### *In vivo* Targeting and MR/US Imaging Performance

**FIGURE 8:**
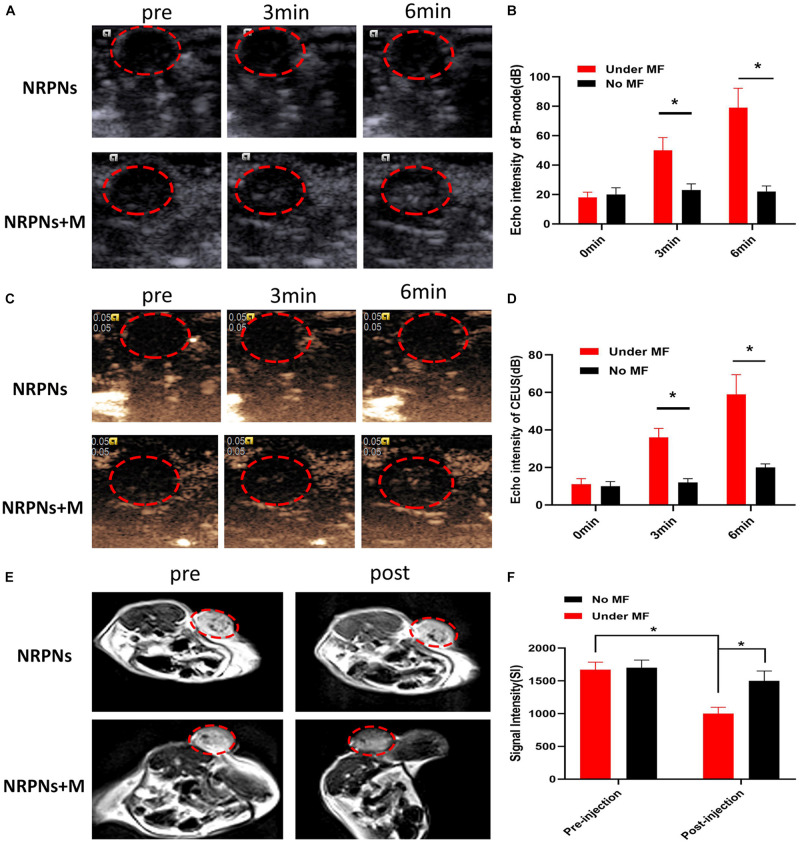
**(A)** *In vivo* B-mode US images and **(B)** the echo intensities of the tumor regions after intravenous injection 24 h of the NRPNs under NIR laser irradiation with different irradiation time. **(C)** *In vivo* CEUS images and **(D)** the echo intensities of the tumor regions after intravenous injection 24 h of the NRPNs under NIR laser irradiation with different irradiation time. **(E)** *In vivo* T2-weighted MR image, and **(F)** the signal intensities of the tumor regions before and after intravenous injection 24 h of the NRPNs. The difference is statistically significant (*p < 0.05).

Due to the excellent *in vitro* vaporization effect induced by laser irradiation, the NRPNs were used as an US enhancement *in vivo*. After magnetic attraction for 8 h, the tumor region of the tumor-bearing mice was irradiated by a NIR laser at 24 h after injection. As shown in Figure 8, there was no or weak enhancement observed in B-mode (Figures 8A,B) and CEUS (Figures 8C,D) in the group without the magnet after NIR-laser irradiation. Some scholars have proven that the an enhanced permeability and retention (EPR) effect promotes the aggregation of NPs in the tumor region (Maeda et al., 2013). However, the amount of the NRPNs engulfed by tumor cells could not produce obvious changes in the US images while there was significant enhancement both in B-mode and CEUS mode as observed in the group with the magnet (^∗^*p* < 0.05). With the help of the magnet, more NRPNs aggregated in the tumor region, demonstrating the excellent targeting ability of the NRPNs. Moreover, the ICG and Fe_3_O_4_ in the NRPNs absorbed the NIR light energy, transferred it to heat, triggered the PFP vaporization process, and induced more echo intensity generation. These results demonstrated the excellent US imaging ability of the NRPNs *in vivo*.

In light of the excellent MRI negative enhancement of the NRPNs *in vitro*, the ability of the NRPNs for use with MR imaging was studied in small animal experiments. T2-weighted MR imaging of the mouse tumor region was obtained after the injection of the NRPNs with and without magnetic targeting. As shown in Figure 8E, an obvious tumor darkening effect was observed in the tumor region at 24 h post injection of the NRPNs with the magnetic targeting field. However, there was no significant change observed in the group in the absence of magnetic targeting at 24 h post-injection of the NRPNs. Moreover, the decreased T2-weighted SI after NP injection with magnetic targeting was significantly lower than that without magnetic targeting (Figure 8F, ^∗^*p* < 0.05). This finding also contributed to the magnetically targeted strategy of the NRPNs.

### *In vivo* Anticancer Efficacy

**FIGURE 9:**
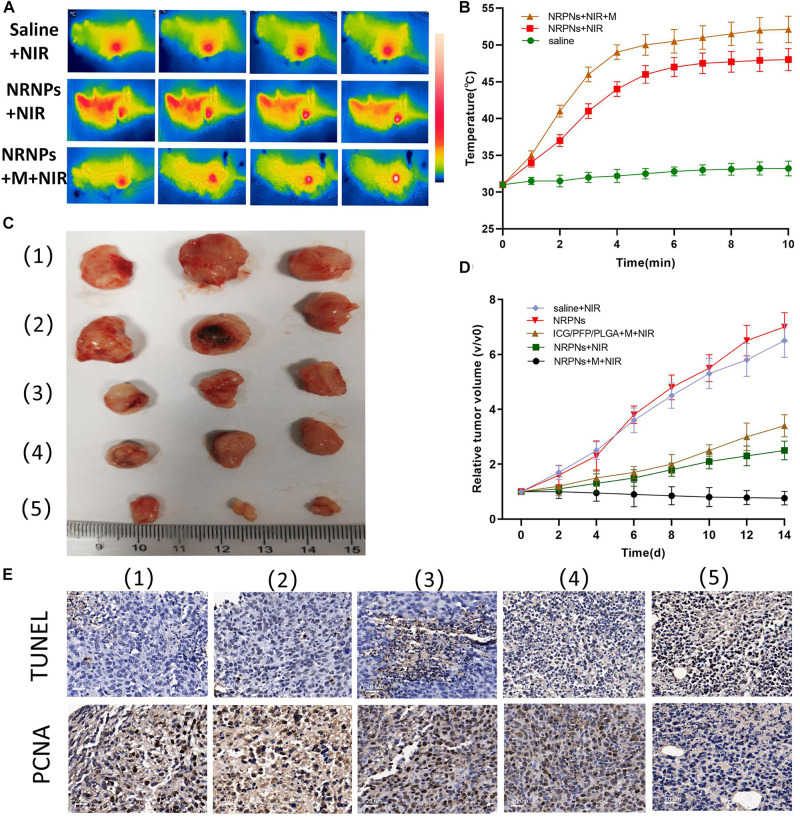
**(A)** Infrared thermal images and **(B)** temperature variation of tumor-bearing mice injected with the saline or NRPNs with or without a magnet under 808 nm laser irradiation for 10 min (1 W/cm^2^). **(C)** Photographs of tumors and **(D)** the relative tumor growth curves during the various treatments after 14 days. **(E)** Microscopy images of TUNEL and PCNA assays of stained tumor tissues at different treatments: (1) Saline, (2) NRPNs, (3) NRPNs + NIR laser, (4) ICG/PFP/PLGA NPs + M + NIR laser and (5) NRPNs + M + NIR laser.

After demonstrating that these NIR responsive phase-shifted nanoparticles can be ueed for MR/US dual model imaging, we then evaluated their PTT effect *in vivo*. Tumor-bearing mice were randomly divided into three groups: (1) saline with laser irradiation, (2) the NRPNs with laser irradiation but without magnetic targeting, and (3) the NRPNs with magnetic targeting and laser irradiation. As shown in Figures 9A,B, the temperature in the tumors of the saline group and the NRPNs without the magnet group increased to 33°C and 48°C, respectively. The temperature increased rapidly to 52°C in the NRPNs with the magnet after laser irradiation, and this temperature is an appropriate temperature for killing tumor cells. Thus, the temperature of the other two groups were lower than that of the NRPNs with the magnet group (^∗^*p* < 0.05), indicating that with the help of the magnetic targeting field, more NPs accumulated into the tumor and then transferred light to heat, which increased to the local temperature.

Then, to evaluate the PTT therapeutic effect of the NRPNs after NIR laser irradiation with a magnet, the tumor growth was observed for 14 days after injection. Tumor-bearing mice were randomly divided into five groups (*n* = 3 for per group): (1) saline with laser irradiation, (2) only the NRPNs, (3) the NRPNs with laser irradiation but without magnetic targeting, (4) ICG/PFP/PLGA NPs with magnetic targeting and laser irradiation and (5) the NRPNs with magnetic targeting and laser irradiation. As shown in Figures 9C,D, in the only NRPNs group (2) and the saline with laser irradiation group (1), the tumor volumes increased significantly, up to seven-fold and 6.5-fold, respectively, compared to day 0, indicating NRPNs or NIR irradiation alone had no antitumor effect. As expected, the PTT effect using NRPNs (3) or ICG/PFP/PLGA NPs (4) showed a good antitumor effect with tumor volumes slowly increasing to 2.5-fold and 3.4-fold, respectively, compared with day 0. Compared with NRPNs without magnet targeting (3), the PTT effect using NRPNs with the magnet targeting group (5) displayed a better antitumor effect with decreased tumor volumes, demonstrating that with the help of magnetic targeting, the NRPNs could more effectively inhibit tumor growth through PTT treatment due to the high accumulation of the NPs in the tumors.

Finally, the PTT efficacy was further evaluated by TUNEL and PCNA immune-histochemical assays. As shown in Figure 9E, the proliferative cells with brown nuclear staining of the TUNEL assay in the NRPNs after laser irradiation group (3) and the ICG/PFP/PLGA with magnetic targeting and laser irradiation group (4) were higher than that in the only NRPNs group (2) and saline with laser irradiation (1), but they were lower than that in the NRPNs with a magnet after laser irradiation group (5). Opposite to the TUNEL expression pattern, the proliferative cells with brown nuclear staining of PCNA expression were the lowest in the NRPNs with a magnet after laser irradiation (5) compared to the other groups. These results showed that PTT combined with the enfolded ICG and Fe_3_O_4_ NPs could inhibit tumor growth and promote tumor apoptosis. With the help of magnetic targeting, PTT was a more efficient anticancer therapy.

## Conclusion

In this study, we developed NRPNs, which improved the MRI and US diagnosis of cancer. The NRPNs showed excellent photothermal transformation capacity, higher ICG loading capacity, good liquid-gas phase transformation capabilities and amazing magnetic response abilities. Irradiation of the tumor site with a laser allowed them assimilate the NIR light rapidly and transform it into enormous heat energy, leading to superior tumor suppression. With a combination of simultaneous MRI and US diagnosis, this kind of multifunctional NPs guided by a magnetic field can be further applied for cancer theranostics.

## Data Availability Statement

The original contributions presented in the study are included in the article/supplementary materials, further inquiries can be directed to the corresponding author/s.

## Ethics Statement

The animal study was reviewed and approved by the Ethics Committee of Central South University.

## Author Contributions

YX and CN: conceptualization and the data curation. YX and WL: methodology. SC, WL, WP, and BH: analysis and investigation. YX: writing original draft preparation. CN: writing-review, editing, and supervision. All authors contributed to the article and approved the submitted version.

## Conflict of Interest

The authors declare that the research was conducted in the absence of any commercial or financial relationships that could be construed as a potential conflict of interest.
